# Sevoflurane Impairs Short-Term Memory by Affecting PSD-95 and AMPA Receptor in the Hippocampus of a Mouse Model

**DOI:** 10.1155/2019/1068260

**Published:** 2019-10-21

**Authors:** Yuan Jiao, Hongwu Fan, Kexin Wang, Shan Lu

**Affiliations:** ^1^Department of Anesthesiology, China-Japan Union Hospital of Jilin University, Changchun 130033, China; ^2^Department of Orthopedics, China-Japan Union Hospital of Jilin University, Changchun 130033, China

## Abstract

**Objective:**

To explore the effects of sevoflurane on the latency and error times of the passive avoidance and levels of PSD-95 and AMPA receptors in the hippocampus. We evaluated the effects of sevoflurane on short-term memory in adult mice and explored the possible mechanism.

**Methods:**

144 Kunming mice (2-3 months, 30-35 g) were randomly divided into two groups A (*n* = 64) and B (*n* = 80) and received the dark-avoidance (DA) and step-down avoidance (SA) tests, respectively. The groups DA and SA were further divided into control (inhaled 40% O_2_ 2 h) and sevoflurane (3.3% sevoflurane and 40% O_2_ 2 h) subgroups. Before inhalation intervention, all mice were trained to be familiar with the Morris water maze (MWM). According to the test points of behavioral indicators, 8 mice were randomly selected from each subgroup at point 12 h (T1), 24 h (T2), 48 h (T3), and 72 h (T4) after inhalation intervention. The step-through latency and error times were measured in 5 min. After the behavioral test, the mice were killed and the tissues of the hippocampus were taken for hematoxylin and eosin (H&E) staining. The expression level of PSD-95 and AMPA receptors in the hippocampus was detected by immunohistochemistry and Western Blot. The changes of synaptic transmission were measured via electrophysiology analysis of hippocampal slices.

**Results:**

The mice in the control subgroups found the platform in a shorter pathway than those in the sevoflurane subgroups during an MWM test. The step-through latency of T1 and T2 in the sevoflurane subgroup was shorter than baseline time, and the error times were increased in 5 min and higher than baseline time when compared with the control subgroup (*P* < 0.05) in the A and B groups. Compared with the control subgroup, the expression level of PSD-95 and AMPA receptors in the hippocampus was decreased at T1 and T2 in the sevoflurane subgroup (*P* < 0.05). The nerve cells were partially swelling. Electrophysiology analysis showed that the levels of PSD-95 and AMPA receptor expression were associated with synaptic transmission.

**Conclusion:**

Sevoflurane impaired short-term memory in adult mice by inhibiting the expression of PSD-95 and AMPA receptors in the hippocampus, which led to the decrease in synaptic transmission.

## 1. Introduction

The inhibition of the central nervous system by general anesthetics can impair memory, leading to consciousness loss and memory defect. Sevoflurane is a halogen inhalation anesthetic with the advantages of rapid action, simple regulation of anesthesia, quick recovery, and less inhibition of circulation and respiratory tract [1, 2]. It is widely used in clinical anesthesia. In recent years, research on sevoflurane has become a hotspot. A large number of basic studies have shown that sevoflurane causes the apoptosis of brain neuron cells in mice [3], impairs short-term learning and memory ability, and leads to learning and memory decline [4], but its impact on memory and whether the effect persists is inconclusive [5]. Although there is no conclusive evidence that sevoflurane is neurotoxic to the human brain, studies have reported that sevoflurane can affect learning, memory, and cognitive ability in the elderly [6] and infants [7]. The patients have been reported to have a decline in memory after anesthesia, recalling past events with varying degrees of impairment, and even early cognitive dysfunction, which develops chronic lesions that can have serious adverse effects on patients' work and life [8]. Many patients and doctors have doubts about the safety of sevoflurane because the actual mechanism of sevoflurane is unclear yet. The recall of preoperative events after anesthesia recovery in surgical patients is a process of memory retrieval. Most studies in the past have reflected the effects of sevoflurane on learning and memory processes [9, 10], but they only reflect their impact on memory retrieval capabilities. The exact molecular mechanism of the effects of sevoflurane on the memory retrieval process remains unclear.

It is well known that learning and memory processes are closely related to synaptic plasticity in the brain and require efficient synaptic transmission and neurotransmitter release [11]. A-Amino-3-hydroxy-5-methyl-4-isoxazole-propionic acid (AMPA) receptor is an ionic glutamate receptor, which mediates rapid excitatory synaptic transmission in the central nervous system, whose dynamic expression in the postsynaptic membrane is associated with long-term potentiation, induction, and depression [12]. AMPA receptor is involved in the regulation of learning and memory activities. Synaptic activity requires the participation of many scaffold proteins on the postsynaptic membrane, while postsynaptic density-95 (PSD-95) is newly discovered protein in the postsynaptic density of glutamatergic synapse [13]. It binds to other proteins through different domains, forms a receptor-signaling molecule-regulatory complex, participates in the formation and maintenance of synaptic connections, and plays an important role in regulating the dynamic activities of the postsynaptic receptor AMPA receptor [14], so it is also closely related to memory. It has been reported that the decreased expression of PSD-95 is associated with impaired spatial working memory [15]. Does sevoflurane have a sustained effect on memory retrieval? Whether the changes in hippocampal PSD-95 and AMPA receptor expression play a role in the effect of sevoflurane on memory retrieval has not been reported. It is of practical significance to explore the effect and possible mechanism of sevoflurane on memory retrieval.

Therefore, this study intends to evaluate the effects of sevoflurane on memory defect by observing the changes in latency and error times of adult mice, the pathological changes of the hippocampus, and the expression of PSD-95 and AMPA receptors in the hippocampus during passive avoidance training. The effects of sevoflurane on short-term memory retrieval and possible mechanism will provide a theoretical basis for memory retrieval disorder in the patients with anesthesia.

## 2. Materials and Methods

### 2.1. Experimental Animal

This study was performed in accordance with the principles of the Basel Declaration and animal research of guidelines from the Animal Research Ethics Committee of China-Japan Union Hospital of Jilin University (Changchun, China). The protocol was also approved by the Animal Research Ethics Committee of China-Japan Union Hospital of Jilin University. 144 Kunming mice, 2-3 months, male and female in half, and 30-35 g, were provided by the Experimental Animal Center of China-Japan Union Hospital of Jilin University (animal production license number: JLCCXK2018-0001). The mice were made familiar with the laboratory environment within 3 d before the experiment, and 5 cages were kept in a separate way. The water was not limited. The mice were entrained in 12 h light and 12 h dark (LD) cycles at 25°C with humidity of 60-80% and allowed free access to water and food.

### 2.2. Animal Grouping

According to the random number table, 160 Kunming mice were randomly divided into two groups A (*n* = 80) and B (*n* = 80) and participated in the dark-avoidance (DA) and step-down avoidance (SA) from a wooden platform [16]. According to different treatments, group DA was randomly divided into the control subgroup (*n* = 40, inhaled 40% O_2_ 2 h) and sevoflurane subgroup (*n* = 40, inhaled 3.3% sevoflurane and 40% O_2_ 2 h). Group SA was divided into 2 subgroups (*n* = 40) in the same manner. According to the different time points of the behavioral experiment test, the mice were stopped for the inhalation of sevoflurane or oxygen intervention after 12 h (T1), 24 h (T2), 48 h (T3), and 72 h (T4). Eight mice were randomly selected for behavioral index determination, and each group of mice was tested only once. Eight mice were selected from the sevoflurane and control subgroups at time points T1, T2, T3, and T4 for the subsequent electrophysiological analysis, respectively. Similarly, the mice in the B group that were stopped inhaling sevoflurane or oxygen intervention for 12 h (T1), 24 h (T2), 48 h (T3), and 72 h (T4) were randomly selected for index measurement.

### 2.3. Morris Water Maze (MWM) Test

MWM consisted of a white cylindrical pool and a transparent plexiglass platform. The pool was 100 cm in diameter and 50 cm high. The water depth in the pool was 30 cm, and the temperature of the water was maintained at 21 ± 2°C with a heater. The pool was divided into four quadrants I, II, III, and IV. A circular transparent escape platform with a diameter of 10 cm and a height of 29 cm was placed at a distance of 20 cm from the wall of the IV quadrant. The platform was covered with scratches to provide animals. The surface was easy to stand, and the pool water needed to be 1 cm below the platform. A camera with a display system was placed above the maze, and the computer automatically tracked the timing and records the swimming trajectory. One day before the experiment, Kunming mice were allowed to swim for 2 min in a pool without a platform to familiarize themselves with the environment. Before inhalation intervention, they were trained 4 times per day for 2 days. The training began by placing the platform in the IV quadrant. After inhalation intervention at T1-T4 time points, the elapsed time was recorded when the mice enter the water and found and climbed the platform (all the limbs climbed the platform). If the mice did not find the platform within 2 min, the experimenter led them to the platform and have them stay on the platform for ten seconds, and the escape latency was recorded as 120 s. The arithmetic mean of the 4 latency was the result to detect the memory ability of Kunming mice.

### 2.4. Anesthesia Test

After the establishment of passive avoidance memory, they were placed in a self-made semiclosed anesthesia box for sevoflurane anesthesia. The anesthesia box was made of plexiglass and had a thin layer of soda lime within a space of 45 cm × 30 cm × 30 cm. The box was continuously introduced with 5% sevoflurane, 40% of carrier gas, and O_2_ flow rate at 2 L/min. When sevoflurane reached the equilibrium concentration 3.3% in the box, the mice were quickly placed. In the box, the mice were given 3.3% sevoflurane for 2 h. An oxygen saturation probe was attached to the abdomen of the mouse to monitor the heart rate, respiratory rate, and pulse oxygen saturation, and the color of the lips and toes of the mouse was observed. The normal pulse oxygen saturation (SPO_2_) of the mice was between 90% and 100%. During anesthesia, mice with SPO_2_ less than 90% for more than 5 min should be eliminated. After inhalation of anesthesia of sevoflurane, the mice continued to receive oxygen for 15 min in the anesthesia box to be restored.

### 2.5. Dark-Avoidance (DA) Test

#### 2.5.1. Training Period (Days 1-2)

The mice were placed in the dark room and made familiar with the environment for 5 min, and the sliding door was then gently opened between the bright room and the dark room, and the time was recorded when the mouse entered the hole. Under the same experimental conditions, the mice were trained 3 times a day, 5 min each time for 2 days to enhance the darkening behavior of mice. Step-through latency (STL) and the shock-box escape latency were recorded using electronic timers. The mice would be eliminated if they did not enter the hole within 5 min. After entering the dark room, the door hole was closed and the mice received electric foot shock. The AC voltage intensity was 32 V for 6 s. After the electric shock, it stayed in the dark room for 10 s.

#### 2.5.2. Treatment Period (Day 4)

After the establishment of the dark memory for 24 h, the STL of all mice and the number of times of entering the dark room within 5 min and error times (ETs) were used as the baseline time. The sevoflurane subgroup inhaled 3.3% sevoflurane and 40% O_2_ for 2 h, while the control group inhaled 40% O_2_ for 2 h.

#### 2.5.3. Test Period (Days 5-7)

Each subgroup performed a dark memory test at the corresponding time point, and STL and ETs were recorded within 5 min. If the mouse did not enter the hole within 5 min, the incubation period was recorded as 300 s, and the number of errors was recorded as 0. The success criteria for the establishment of DA memory are as follows: STL > 120 s in the baseline score, and the occurrence of forgetting was STL < 30 s during the test period.

### 2.6. Step-Down Avoidance (SA) Test

#### 2.6.1. Training Period (Day 1)

The mice were placed on the platform in the jumping box for 3 min. The copper grid was charged, the voltage was 100 V, and the current was 3 mA. When the mouse jumped off the platform and received an electric shock, it would jump back to the platform to avoid the electric shock. They jumped off the platform again and then jumped back to the platform after being shocked. The mice learned how to escape the shock and are trained for 5 min. After training for 24 h (Day 2), the step-down latency (SDL) and ETs were recorded at baseline time.

#### 2.6.2. Treatment Period (Day 2)

The mice in the sevoflurane subgroup inhaled 3.3% sevoflurane and 40% O_2_ for 2 h, and the control subgroup inhaled 40% O_2_ for 2 h.

#### 2.6.3. Test Period (Days 3-5)

The SA test was repeated, and SDL and ETs within 5 min were recorded. If the mouse did not jump off the platform within 5 min, the latency of the step was recorded as 300 s, and the number of errors was recorded as 0.

### 2.7. Hematoxylin-Eosin (H&E) Stain

After the behavioral test at each time point, 5 mice in each subgroup were randomly selected for hippocampal tissue preparation. 10% chloral hydrate (0.4 mL/100 g) was intraperitoneally injected. After successful anesthesia, the mice were placed on the wooden board in the supine position. The skin was cut through the sternum, and then, the xiphoid was lifted and the chest cavity was cut. The perfusion needle was then inserted into the left ventricle from the apex while the right atrial appendage was cut. Precooled PBS solution was pushed, and the color of the perfusion to the lungs and liver becomes grayish white, and reperfusion of 4% paraformaldehyde was made until the limbs were stiff. The head of the mouse was cut off, the skull was cut to expose the skull, the skull was separated, and the brain was removed gently. The brain tissue was then fixed in neutral formalin solution for 24 h, then dehydrated by alcohol gradient, and finally paraffin-embedded for H&E staining. Paraffin-embedded hippocampal tissues were cut into 3 *μ*m thick and stained with H&E. The pathological changes in the hippocampus were observed under a light microscope. The number and volume of hippocampus cells were measured by using ImageJ software (NIH, Bethesda, USA).

### 2.8. Western Blot Analysis

5 mg hippocampus tissues were placed into an EP tube, and the lyase was added, homogenized on ice for 15 min with an electric tissue homogenizer. The lysate was centrifuged at 12000 × *g* at 4°C for 10 min, and the supernatant was collected. The protein was separated by using SDS-PAGE and transferred to a PVDF membrane. After the transfer was completed, the strip was taken out and immersed in the blocking solution and blocked at room temperature for 2 h on a shaker. Then, PSD95 primary antibody (dilution 1 : 500), AMPA receptor primary antibody (dilution 1 : 1000), and *β*-actin (dilution 1 : 2000) were added and incubated at 4°C overnight. The membrane was washed with TBST for 10 min and washed 3 times, and then, HRP-conjugated goat anti-rabbit IgG H&L secondary antibody (dilution 1 : 8000) was added and incubated for 1 h at room temperature on a shaker. The strip was rinsed with TBST for 10 min, repeat 3 times, and ECL was added. The protein bands were visualized using the Odyssey CLx Infrared Imaging System (LI-COR, Nebraska, USA).

### 2.9. Electrophysiology Analysis of Hippocampal Slices

Eight mice from the sevoflurane subgroup and 8 mice from the control subgroup were chosen for electrophysiological study. According to a previous report [17], the mice were cervically dislocated and decapitated from T1 to T4 time points, respectively, and their brains were quickly removed immediately and placed in cold oxygenated buffer (in mM: NaCl, 120; KCl, 3; MgCl_2_, 2; CaCl_2_, 2; NaH_2_PO_4_, 1.2; NaHCO_3_, 23; and glucose, 11). Hippocampi were separated and cut into 400 *μ*m thick transverse slices by using a McIlwain tissue chopper (Westbury, NY, USA). The slices were transferred to a submerged recording chamber or stored in a chamber for 1 h before recording. Evoked responses were produced by stimulating Schaffer collateral, commissural, and perforant path fibers, respectively. Long-term potential (LTP) was produced by increasing the concentration of Mg^2+^ and Ca^2+^ to 4 mM with 0.1 mM picrotoxin (catalog: P1675; Sigma-Aldrich). After a-quarter baseline recording (30 s intervals) for both pathways, LTP was induced by 6 trains (3 s interinterval) of 20 pulses at 100 Hz, which was 1.5-fold of the baseline intensity. In control slices, the tetanus was replaced by a low-frequency train with the same stimuli (0.1 Hz) for a quarter. Thereafter, stimulating strength was regulated to the same excitatory postsynaptic potential (EPSP) in the potentiated slice. Thus, it was the same for the number of higher-intensity stimuli and responses in the posttetanus period. The whole experiment was performed at 25°C.

### 2.10. Statistical Analysis

All experimental data were analyzed by SPSS 22.0 software. The normal distribution of measurement data was expressed as the mean ± S.D.(standard deviation), and the nonnormal distribution measurement data was expressed as the median (interquartile range). Two independent samples were used for comparison between groups; the comparison between the basic time of each time point and the test period time was performed by paired test analysis; *P* < 0.05 was considered statistically significant.

## 3. Results

### 3.1. Sevoflurane Caused Memory Loss

**Figure 1:**
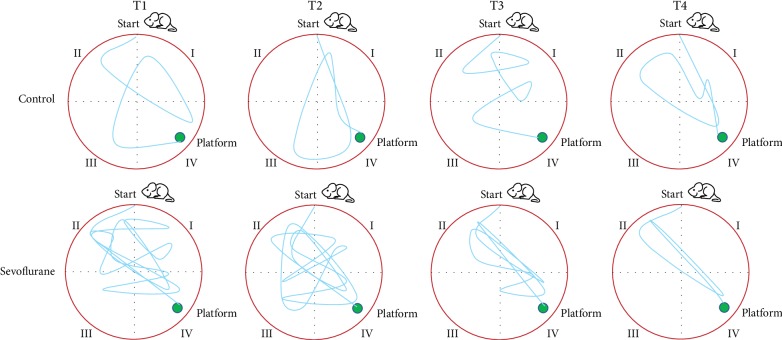
Morris water maze (MWM) test between the control and sevoflurane groups. MWM was divided into I, II, III, and IV quadrants, and a platform was placed in the IV quadrant. According to the different time points of the behavioral experiment test, the mice were stopped for the inhalation of sevoflurane or oxygen after 12 h (T1), 24 h (T2), 48 h (T3), and 72 h (T4).

The MWM test showed that the mice in the sevoflurane subgroups swam a longer pathway than that in the control subgroup to find the platform at T1 and T2 time points (Figure 1). The length of the pathway was shortened in the sevoflurane subgroup when the test was performed at T3 and T4 time points while there was little change for control subgroups. The average latency of mice in the sevoflurane subgroups was longer than that in the control group while the times for crossing the platform and residence time in the target quadrant in the control subgroup were longer than those in the sevoflurane subgroups at T1 and T2 time points (Table 1, *P* < 0.05). The statistical difference for these parameters except for the times for crossing the platform was insignificant at T3 and T4 time points (Table 1, *P* > 0.05). The results suggested that sevoflurane treatment impaired short-term memory until after T2 time point.

### 3.2. Sevoflurane Reduced STL and SDL and Increased ETs

The memory of DA and SA was successfully established, and no mouse was withdrawn. There was no significant difference in the baseline time between the two subgroups in terms of latency and number of errors (*P* > 0.05). Compared with the control subgroup, STL and SDL were shortened and ETs increased in T1 and T2 in the sevoflurane subgroup, in 5 min (*P* < 0.05). Compared with the baseline time, there was no significant difference in the test time between the control group and the ETs within 5 min (*P* < 0.05) (Table 2).

Compared with the control group, SDL was shortened in the sevoflurane group (Table 3, *P* < 0.05), and the difference of ETs in 5 min was statistically significant (Table 3, *P* < 0.05), while T2, T3, and T4 were different, but the statistical difference was insignificant. Compared with the baseline results, there was no significant difference in the test time between the SDL and the 5 min ETs in the control group (*P* > 0.05). The SDL in the sevoflurane group was shortened, and the ETs increased in 5 min. The difference was significant (*P* < 0.05).

### 3.3. Sevoflurane Impaired Nerve Cells in the Hippocampus

**Figure 2:**
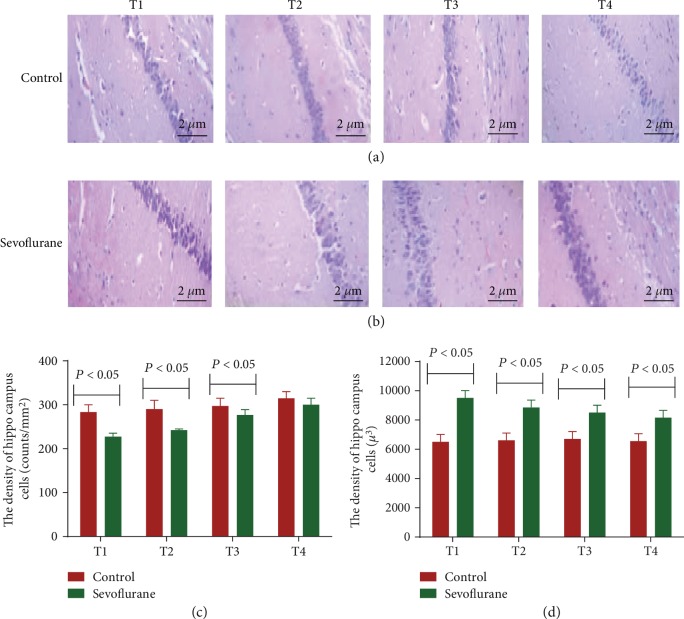
H&E stain analysis of the nerve cells in the hippocampus between the control and sevoflurane groups. According to the different time points of the behavioral experiment test, the mice were stopped for the inhalation of sevoflurane or oxygen after 12 h (T1), 24 h (T2), 48 h (T3), and 72 h (T4).

Under a light microscope, the pyramidal cells in the hippocampus of the control group were closely arranged and structurally intact. The cytoplasm and nucleus of the cells were evenly stained; the morphology of nerve cells and glial cells was normal from T1 to T4 time points (Figure 2(a)). In the sevoflurane group, some nerve cells were swollen in the hippocampus, and the degree of damage gradually decreased with time, but no obvious apoptosis was observed from T1 to T4 time points (Figure 2(b)). The changes for the density of hippocampus cells were little from T1 to T4 time points in the control group (Figure 2(c)) and increased in the sevoflurane group (Figure 2(d)). The difference was significant from T1 to T3 time points (*P* < 0.05). The volume of hippocampus cells changed little in the control group while it was reduced in the sevoflurane group, but the volume of the cells in the control group was still lower in the control group than in the sevoflurane group from T1 to T4 time points (Figure 2(d), *P* < 0.05).

### 3.4. Sevoflurane Reduced Relative Protein Levels of PSD-95 and AMPA Receptor

**Figure 3:**
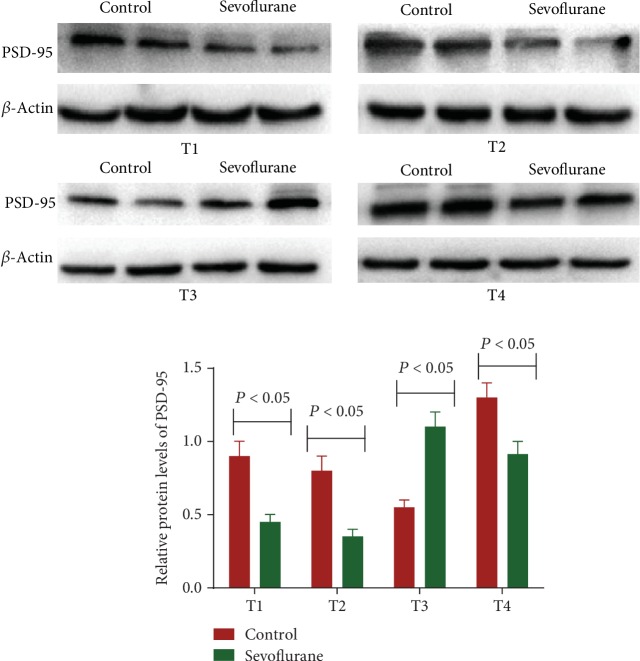
Western Blot analysis of relative protein levels of PSD-95. The mice were stopped for the inhalation of sevoflurane or oxygen after 12 h (T1), 24 h (T2), 48 h (T3), and 72 h (T4). *n* = 8 for each group, and the statistical difference was significant if *P* < 0.05 vs. the control group.

Western Blot showed that relative protein levels of PSD-95 in the hippocampus in the sevoflurane subgroup were lower than that in the control subgroup during T1, T2, and T4 (Figure 3, *P* < 0.05). In contrast, the levels in the sevoflurane group were higher than that in the control group during T3 (Figure 3). The sevoflurane treatment will cause the reduction in the level of PSD-95 in a short time. However, with the time going, the effects of sevoflurane are reducing, and the level of PSD-95 is recovering. The increase is not stable, and we guess that the increase reached the top at T3 time point. Further work is needed to confirm the supposal.

**Figure 4:**
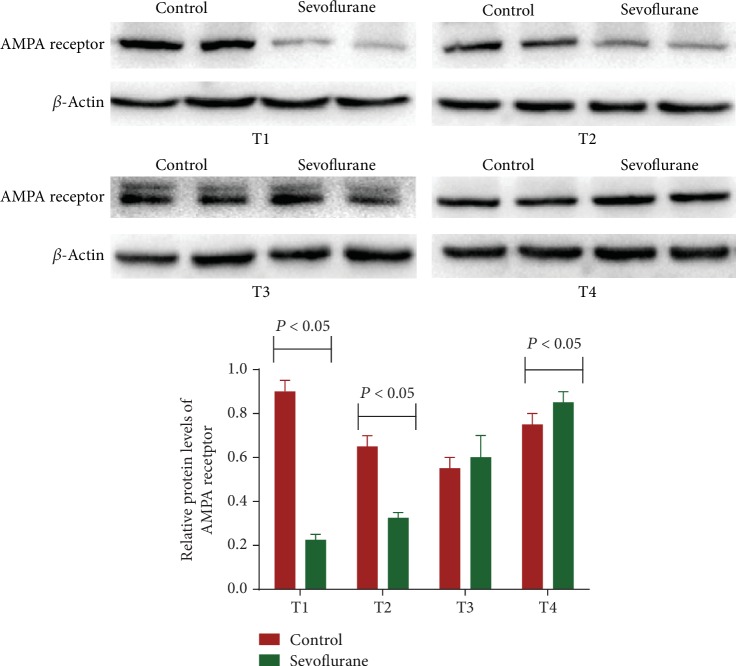
Western Blot analysis of the effects of sevoflurane on relative protein levels of AMPA receptor. According to the different time points of the behavioral experiment test, the mice were stopped for the inhalation of sevoflurane or oxygen after 12 h (T1), 24 h (T2), 48 h (T3), and 72 h (T4). *n* = 8 for each group, and the statistical difference was significant if *P* < 0.05 vs. the control group.

In the similar cases, relative protein levels of AMPA receptor in the hippocampus in the sevoflurane group were lower than that in the control group during T1 and T2 (Figure 4, *P* < 0.05). In contrast, the levels in the sevoflurane subgroup were higher than that in the control subgroup during T4 (Figure 4). The results suggested that sevoflurane reduced relative protein levels of PSD-95 and AMPA receptor in a short term.

### 3.5. PSD-95 and AMPA Receptor Expression Was Associated with Synaptic Transmission

**Figure 5:**
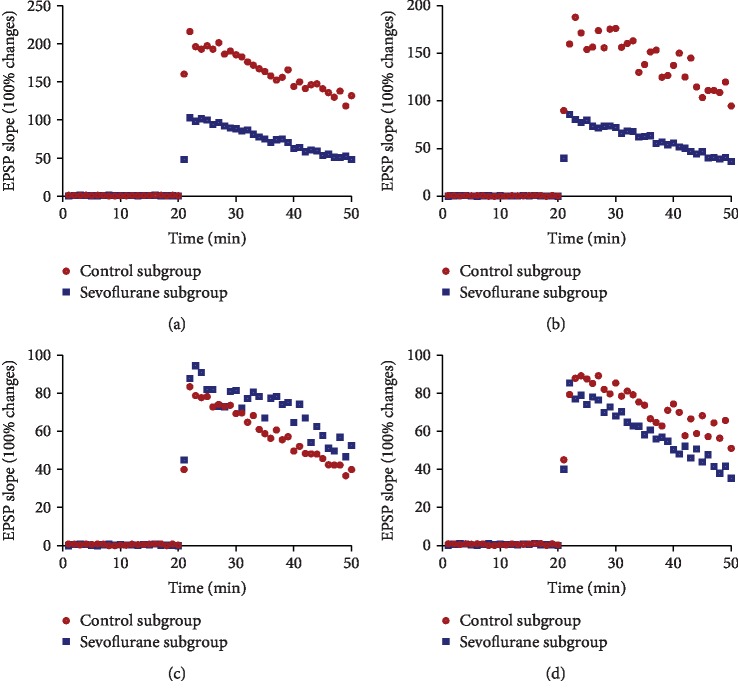
Time-course of long-term potentiation (LTP) of hippocampal slices. The responses were recorded in hippocampal tissue to test stimuli delivered to the perforant path or Schaffer commissural fibers. Tetanic stimulation was performed at 20 min. According to the different time points of the behavioral experiment test, the mice were stopped for the inhalation of sevoflurane or oxygen after 12 h (T1) (a), 24 h (T2) (b), 48 h (T3) (c), and 72 h (T4) (d).

The LTP was measured exclusively on hippocampal slices (Figure 5). The present findings demonstrated that average LTP in the control subgroup was higher than that in the sevoflurane subgroup at time points T1 (Figure 5(a)) and T2 (Figure 5(b)) (*P* < 0.05). The reverse results were found at T3 time point (Figure 5(c)). On the other hand, the levels of PSD-95 in the control subgroup were higher than that in the sevoflurane subgroups at time point T4 (Figure 3) while the levels of AMPA receptor in the control subgroup were lower than that in the sevoflurane subgroups at time point T4 (Figure 4). Meanwhile, the levels of PSD-95 (Figure 5(a)) and AMPA receptor (Figure 5(b)) in the control subgroup were also higher than that in the sevoflurane subgroup. At the time point T3, the levels of PSD-95 and AMPA receptor in the control subgroup were also lower than that in the sevoflurane subgroup (Figure 5(c)). The changing trend was consistent with the level changes of PSD-95 and AMPA receptor (Figures 3 and 4). The results suggested that PSD-95 and AMPA receptor expression was associated with synaptic transmission. The difference for the levels of LTP was reduced ((Figure 5(d)).

## 4. Discussion

Sevoflurane is one of the most widely used inhaled anesthetics in the clinical practice. In recent years, there have been a lot of studies to prove that sevoflurane has neurotoxic effects on the developmental and senile brains [18, 19], which make people have doubts about the safety of sevoflurane. The recall of preoperative events after anesthesia recovery in surgical patients is a retrieving process that acquires memory. At present, there are a few studies on the effects of anesthetic drugs on the memory retrieval process [20, 21], but the exact molecular mechanism remains widely unknown. Therefore, this study explored the effect of sevoflurane on memory retrieval and its possible mechanism of action and the changes of memory biomarkers, which has certain reference value for safe clinical application of these drugs. In recent years, many scholars have studied the effects of sevoflurane on learning and memory in adult rats, but there is no certain conclusion. Xie et al. found that inhalation of 3% sevoflurane for 2 h could not only lead to short-term cognitive dysfunction in adult rats but also lead to reversible long-term cognitive dysfunction [22]. In contrast, Xie et al. found that 0.65 MAC sevoflurane anesthesia had no significant effect on short-term learning and memory ability of adult rats [23]. Haseneder et al. found that 1 MAC sevoflurane anesthesia for 2 h could improve the cognitive ability of adult mice [24]. Our study of behavioral experiments showed that inhalation of 3.3% sevoflurane for 2 h had a short-term inhibitory effect on memory retrieval in passive avoidance experiments in adult mice [24].

Memory retrieval is the basis for generating cognitive ability, and the process is very complicated. From the perspective of information processing, the dynamic process of memory can be divided into three separate stages: encoding, storage, and retrieval [25]. The mechanism is very complicated, so the differences in factors such as drug concentration, timing of administration, duration of maintenance, behavioral test, and animal species can cause differences in the results of these studies. Studies have shown that, unlike humans, the MAC value of sevoflurane in adult mice is 2.6% [26], and the minimum effective sevoflurane concentration required for clinical surgery is 1.2 MAC-1.3 MAC [27]. The purpose of this experiment was to observe the effect of inhaled clinical anesthetic concentration of sevoflurane on the memory retrieval process of adult mice, so 1.3 MAC (about 3.3%) was used as the target concentration. The MWM test demonstrated that sevoflurane intervention impaired short-term memory in the mice at T1 and T2 time points (Table 1 and Figure 1, *P* < 0.05). In the past, in order to study the effects of drugs on learning and memory, most scholars evaluated the effects of drugs on learning ability and memory acquisition. The purpose of this study was to observe the effect of sevoflurane on the ability to extract memory. Therefore, the timing of administration was set after the passive avoidance memory was successfully established in mice, and the passive avoidance memory of mice was tested at different time points after anesthesia.

For the behavioral detection selected in this study, DA and SA tests are recognized as the main behavioral experiments for studying the dynamic process of learning and memory, sensitive to memory processes and especially memory reproduction [28]. The short-term test may affect the results, and each test time was set up with a corresponding control. Through preexperiment, we found that there was no significant difference in autonomic activity between 12 h after stopping inhalation of 3.3% sevoflurane and 12 h after stopping oxygen inhalation, indicating that the difference in latency and number of errors in passive avoidance experiments can directly reflect memory ability, not affected by the residual effects of narcotic drugs.

In this study, DA and SA tests showed that STL and SDL were significantly prolonged, and ETs were significantly reduced within 5 min. The basic results before inhaling sevoflurane indicated that the mice had successfully obtained passive avoidance memory. Combined with the results of behavioral tests, this study found that mice had a forgotten phenomenon in passive avoidance memory within 24 h after stopping inhalation of sevoflurane, which was most obvious at 12 h, and disappeared 48 h and 72 h after stopping inhalation of sevoflurane. Avoiding memory recovery, sevoflurane did not affect the memory storage process, and the forgetting phenomenon within 24 h was a memory retrieval disorder.

Studies have shown that the hippocampus is closely related to fear memory [29]. Therefore, the hippocampus was used to observe whether sevoflurane caused pathological changes, especially apoptosis. The effect of sevoflurane on the expression of memory-related proteins was measured in the hippocampus. Western Blot analysis showed that there was no obvious pyramidal cell apoptosis in the hippocampus after inhalation of sevoflurane, but some nerve cells were swollen within 24 h, and the degree of damage gradually decreased with time going. Sevoflurane did not cause serious irreversible damage to nerve cells.

It is well known that the hippocampus is a brain region closely related to learning and memory [30]. Synaptic plasticity is currently considered to be the main mechanism of learning and memory. It is dynamic neural activity during the process of transmitting information between two neurons. The regulation of synaptic plasticity is reflected in the regulation of synaptic morphological plasticity and the expression of various receptors and proteins on the synaptic membrane [31]. The AMPA receptor is one of the ionotropic glutamate receptors and mediates the excitatory postsynaptic currents [32]. PSD-95 is a major scaffold protein isolated from the postsynaptic dense band structure [33]. PSD-95 regulates N-methyl-D-aspartate (NMDA) receptor function, endocytosis, and transformation of AMPA receptors, and links cytoskeleton and postsynaptic plasma membrane, and is involved in signal transduction [33]. Studies have confirmed that activation of memory retrieval requires sustained protein synthesis to act on NMDA receptor-mediated AMPA receptor transport [34]. PSD-95 acts as an essential molecule for AMPA receptor anchoring on the postsynaptic density [14]. In mature synapses, overexpression of PSD-95 promoted dendritic maturation and AMPA receptor membrane metastasis [35]. Our results showed that the expression of PSD-95 and AMPA receptors in the hippocampus of mice was downregulated after inhalation of sevoflurane, but this effect only occurred within 24 h after stopping inhalation of sevoflurane, and the expression of PSD-95 and AMPA receptors in the hippocampus returned to normal after 48 h. The trend of change was consistent with behavioral findings, suggesting that sevoflurane inhibited the expression of PSD-95 and AMPA receptors in the hippocampus, possibly by inhibiting postsynaptic signaling, leading to a reduction in effective synaptic transmission, leading to memory retrieval disorders, but this effect could not persist.

The present findings indicated that PSD-95 and AMPA receptor expression was associated with synaptic transmission. The results were accordant with the previous report. PSD-95, an excitatory postsynaptic scaffolding protein, is a prominent organizer of the postsynaptic density [36, 37]. AMPA regulates postsynaptic conductance by activating NMDA receptor, which mediates AMPA receptor transport [34], and the increase in the frequency of AMPA receptor mediates spontaneous EPSC [38].

There are still some limitations in this experiment. First, the brains of humans and animals are significantly different in structure and development. Human brain volume and number of neurons are greater than those of rodents. Due to the different life cycle, the onset and duration of synaptic development in human brains are very different from those in rodents. Secondly, inhalation anesthetics are rarely used alone in clinical anesthesia, and analgesics, muscle relaxants, sedatives, and vasoactive drugs are mixed, and the interaction among drugs is complicated. And clinically, patients still have tension, pain, basic diseases, blood loss, and other factors during the perioperative period, which cannot be simulated in animal experiments. Therefore, our experimental research is unlikely to be completely consistent with the clinical state, so the conclusions obtained cannot be directly applied to the clinic. Third, under different inhalation concentrations and different durations of inhalation, sevoflurane has the same effect on memory retrieval in adult mice. Further work is needed to confirm the present conclusion.

## 5. Conclusion

Sevoflurane inhibited short-term memory retrieval in adult mice and may be associated with the inhibition of PSD-95 and AMPA receptor in the hippocampus, leading to a decrease in effective synaptic transmission.

## Figures and Tables

**Table 1:** Morris water maze between control and sevoflurane subgroups (*n* = 8).

	Control	Sevoflurane	*P* values
Latency time (seconds)			
T1	42.61 ± 5.77	59.73 ± 9.35	0.001
T2	41.95 ± 10.44	55.18 ± 9.88	0.001
T3	43.31 ± 11.36	47.37 ± 10.53	0.055
T4	44.26 ± 11.89	46.57 ± 12.01	0.129
Residence time in the targeted quadrant (seconds)			
T1	49.64 ± 5.53	39.12 ± 8.21	0.007
T2	47.94 ± 9.97	42.98 ± 9.25	0.026
T3	46.58 ± 10.69	47.72 ± 9.69	0.285
T4	48.85 ± 11.32	49.62 ± 10.21	0.394
Number of crossing platform			
T1	5.58 ± 1.08	3.43 ± 2.10	0.001
T2	5.30 ± 1.78	4.17 ± 2.17	0.001
T3	5.89 ± 2.14	5.01 ± 2.52	0.045
T4	6.04 ± 2.53	5.82 ± 2.68	0.652

**Table 2:** Effect of sevoflurane on indicators in the step-through latency test of mice (*n* = 8).

Subgroups	Time	Baseline		Test period	
		Latency (s, *x* ± *s*)	Number of errors (times, domain 0)	Latency (s, *x* ± *s*)	Number of errors (times, domain 0)
Sevoflurane	T1	299.998 ± 32.003	0 (0)	36.67 ± 29.501^ab^	2.50 (2.75)^ab^
	T2	268.075 ± 44.572	0 (0)	64.23 ± 100.43^ab^	3.25 (4.88)^ab^
	T3	255.998 ± 31.863	0 (0.75)	216.33 ± 31.86	0.50 (2.00)
	T4	188.931 ± 128.959	0 (0.75)	133.91 ± 111.52	0.50 (1.00)
Control	T1	265.148 ± 98.575	0 (0)	298.31 ± 44.30	0 (0.75)
	T2	288.955 ± 42.021	0 (0)	261.83 ± 65.84	0 (1.25)
	T3	299.361 ± 1.432	0 (0)	207.27 ± 57.31	0 (0.75)
	T4	235.493 ± 120.195	0 (0)	204.89 ± 125.52	0 (0.75)

Note: ^a^*P* < 0.05*vs*. the control group and ^b^*P* < 0.05*vs*. the baseline time.

**Table 3:** Effect of sevoflurane on latency and error times in the step-down latency test of mice (*n* = 10).

Groups	Time	Baseline time		Test period time	
		Latency (s, *x* ± *s*)	Number of errors (times, domain 0)	Latency (s, *x* ± *s*)	Number of errors (times, domain 0)
Sevoflurane	T1	180.5 (100.2)	1.0 (1.0)	37.5 (66.0)^ab^	3.0 (3.5)^ab^
	T2	211.5 (79.5)	1.5 (1.0)	153.5 (168.5)	1.5 (2.8)
	T3	274.0 (53.8)	1.0 (1.0)	297.5 (47.0)	1.0 (0.0)
	T4	262.0 (104.8)	l.0 (l.0)	290.0 (105.0)	1.0 (0.0)
Control	T1	253.5 (105.8)	1.5 (1.8)	164.0 (136.5)	1.0 (1.0)
	T2	253.0 (63.5)	1.0 (0.8)	173.0 (265.0)	1.0 (1.5)
	T3	260.5 (78.8)	1.5 (1.0)	294.5 (102.0)	1.0 (0.0)
	T4	254.0 (97.0)	1.0 (1.0)	296.0 (9.75)	1.0 (0.8)

Note: ^a^*P* < 0.05*vs*. the control group and ^b^*P* < 0.05*vs*. the baseline time.

## Data Availability

The data for the current study are available from the corresponding author upon reasonable request.
